# ATP synthase ecto-α-subunit: a novel therapeutic target for breast cancer

**DOI:** 10.1186/1479-5876-9-211

**Published:** 2011-12-08

**Authors:** Jian Pan, Li-Chao Sun, Yan-Fang Tao, Zhuan Zhou, Xiao-Li Du, Liang Peng, Xing Feng, Jian Wang, Yi-Ping Li, Ling Liu, Shui-Yan Wu, Yan-Lan Zhang, Shao-Yan Hu, Wen-Li Zhao, Xue-Ming Zhu, Guo-Liang Lou, Jian Ni

**Affiliations:** 1 Department of Hematology and Oncology, Children's Hospital of Soochow University, Suzhou, 215003, China; 2 State Key Laboratory of Molecular Oncology, Cancer Institute (Hospital), Peking Union Medical College, Chinese Academy of Medical Sciences, Beijing 100021, China; 3 Hillman Cancer Center Lab, Department of Pathology, Pittsburgh University, G21 5117 Center Ave. Pittsburgh, PA 15206, USA; 4 Laboratory of Cellular Oncology, National Cancer Institute, NIH, Building 37, Room 4112, Bethesda, MD 20892, USA; 5 Institute of Clinical Medical Science, China-Japan Friendship Hospital, Beijing, China; 6 Translational Research Center, Chang Hai Hospital, the Second Military Medical University, Shanghai, China; 7 Translational Research Center, Second Hospital, The Second Clinical School, Nanjing Medical University, Nanjing, China

**Keywords:** Two-dimensional liquid phase chromatographic fractionation, ATP synthase α-subunit, Tissue microarray, breast cancer, monoclonal antibody

## Abstract

**Background:**

Treatment failure for breast cancer is frequently due to lymph node metastasis and invasion to neighboring organs. The aim of the present study was to investigate invasion- and metastasis-related genes in breast cancer cells *in vitro *and *in vivo*. Identification of new targets will facilitate the developmental pace of new techniques in screening and early diagnosis. Improved abilities to predict progression and metastasis, therapeutic response and toxicity will help to increase survival of breast cancer patients.

**Methods:**

Differential protein expression in two breast cancer cell lines, one with high and the other with low metastatic potential, was analyzed using two-dimensional liquid phase chromatographic fractionation (Proteome Lab PF 2D system) followed by matrix-assisted laser desorption/time-of-flight mass spectrometry (MALDI-TOF/MS).

**Results:**

Up regulation of α-subunit of ATP synthase was identified in high metastatic cells compared with low metastatic cells. Immunohistochemical analysis of 168 human breast cancer specimens on tissue microarrays revealed a high frequency of ATP synthase α-subunit expression in breast cancer (94.6%) compared to normal (21.2%) and atypical hyperplasia (23%) breast tissues. Levels of ATP synthase expression levels strongly correlated with large tumor size, poor tumor differentiation and advanced tumor stages (*P *< 0.05). ATP synthase α-subunit over-expression was detected on the surface of a highly invasive breast cancer cell line. An antibody against the ATP synthase α-subunit inhibited proliferation, migration and invasion in these breast cancer cells but not that of a non-tumor derived breast cell line.

**Conclusions:**

Over-expression of ATP synthase α-subunit may be involved in the progression and metastasis of breast cancer, perhaps representing a potential biomarker for diagnosis, prognosis and a therapeutic target for breast cancer. This finding of this study will help us to better understand the molecular mechanism of tumor metastasis and to improve the screening, diagnosis, as well as prognosis and/or prediction of responses to therapy for breast cancer.

## Background

Breast cancer is one of the most frequently diagnosed and deadly cancers, with an estimated incidence of 7.6-9.1/10 000 inhabitants worldwide per year [1]. For some decades, studies of molecular alterations in tumors have successfully elucidated some mechanisms of mammary carcinogenesis, progression and metastasis, and identified key genes such as ERBB2, TP53, CCND1, BRCA1 and BRCA2 [2,3]. Although the survival of patients has increased over the last decades due to screening programs and considerable progress in post-operative adjuvant systemic therapies (hormone therapy and chemotherapy) targeting hormonal receptors and the ERBB2/HER2 receptor [1,4,5], many patient deaths still occur after metastatic relapse. Prognostic markers currently accepted for clinical use, such as nodal status, tumor size, histological grade, steroid receptor status and others do not adequately identify patients at an early stage, increasing the risk of progression and metastasis [6]. Therefore, additional prognostic biomarkers for the clinical management of breast cancer patients are needed.

High-throughput genomic and proteomic techniques provide unprecedented opportunities to tackle the complexity of breast cancer [3,7,8]. A combination of biomarkers will likely be more sensitive and specific than a single biomarker to reflect the true heterogeneity of disease, more reliable for screening, diagnosis, prognosis and prediction of therapeutic responses, and more useful for finding new therapeutic targets [9]. Among the currently available techniques, proteomic analysis by two-dimensional mass spectrometry (2DE-MS) permits the screening of thousands of modified or unmodified proteins simultaneously, becoming increasingly popular for identifying biomarkers for early detection, classification and prognosis of tumors, as well as pinpointing targets for improved treatment outcomes [8,10]. A relatively newcomer to analytical proteomics is the commercial instrument PF 2D from Beckman Coulter, which uses chromatographic focusing to separate intact proteins in the first dimension by pI (from 8.5 to 4.0) and, in the second dimension, by reversed phase chromatography, which separates proteins based on hydrophobicity. Thus, the precise detection of isoforms and/or proteins with post-translational modifications that alter the pI and/or hydrophobicity is enhanced.

In the present study, we conducted proteomic analysis on two breast carcinoma cell lines, MCF-7-H and MCF-7, with different metastasis potentials, by 2D liquid phase chromatographic fractionation using the PF 2D system [11,12], followed by matrix-assisted laser desorption/time-of-flight mass spectrometry (MALDI-TOF/MS), tissue microarray (TMA), immunological and functional analysis. One of the highly over-expressed proteins was identified as the α-subunit of ATP synthase. ATP synthase is responsible for ATP production in oxidative phosphorylation and can work in reverse as a proton pumping ATPase [13,14]. ATP synthase expression is believed to be localized exclusively to mitochondria where it generates most cellular ATP. However, ATP synthase components have recently been identified as cell-surface receptors for apparently unrelated ligands in the course of studies carried out on angiogenesis [15-17], lipoprotein metabolism [18], innate immunity [19], hypertension [20] or regulation of food intake [21] by immunofluorescence, biochemistry and proteomics analyses [15]. Its molecular mechanism, function and significance have not been fully established. We detected the expression of ATP synthase α-subunit protein by immunohistochemistry (IHC) in different human tumor samples, including breast cancer, hepatocellular carcinoma, colon cancer and prostate cancer. As ATP synthase α-subunit was highly over-expressed in 94.6% of breast cancer samples tested, the present study is focused on the expression, functional implication and potential involvement of ATP synthase in the progression and metastasis of breast cancer.

## Methods

### Cell culture conditions

The breast cancer cell line MCF-7 (a gift from Zhi-Hua Yang, Chinese Academy of Medical Sciences, Peking Union Medical College, Beijing, China) was cultured in RPMI 1640 supplemented with 10% fetal bovine serum (FBS). The highly invasive breast cancer cell line MDA-MB-231 and the immortalized human breast epithelial cell line MCF-10F were purchased from American Type Culture Collection (ATCC, Manassas, VA). MDA-MB-231 cells were cultured in DMEM medium supplemented with 10% FBS. MCF-10F cells were cultured in Ham's F12 medium supplemented with 10% FBS and 20 μg/ml of epidermal growth factor. All cells were maintained at 37°C and 5% CO_2_.

### Selection of a invasive subline from MCF-7 cells

MCF-7 cells were seeded on a Matrigel (Becton Dickinson, Franklin Lakes, NJ) coated, 8 μm-pore transwell (Costar, Cambridge, MA)[12,22,23]. Twenty-four hours later, cells that had invaded to the other side of the trans well membrane were collected, expanded and then re-seeded into another Matrigel coated trans well. Such selection rounds for highly invasive cells were repeated six times, resulting in a highly invasive subline designated as MCF-7-H.

### 2-D liquid chromatography and MALDI-TOF/MS analysis

The Proteome Lab PF 2D two-dimentional liquid chromatography system (Beckman Coulter, CA, USA) consists of 1st dimension chromatofocusing separation based on pI and 2nd dimension reverse-phase chromatography separation based on hydrophobicity. Chromatofocusing was carried out on the chromatofocusing column by mixing two buffers with different pH levels, Starting Buffer (pH 8.5) and Elution Buffer (pH 4.0), to create a linear pH gradient from 8.5 to 4.0, which was followed by wash buffer (1 M NaCl). Cell lysates (2 mg) of MCF-7-H and MCF-7 with different metastasis potentials were injected onto the chromatofocusing column equilibrated for 130 min at 0.2 ml/min with the proprietary starting buffer including urea and a reducing agent at pH 8.5. Fractions were collected at 0.3-pH intervals, and each fraction (200-500 μl) was sequentially analyzed by reverse-phase HPLC. Proteins were separated at a flow rate of 0.75 ml/min on a non-porous C18 reverse phase column using 3.33% B/min linear gradient in which solvent A was 0.1% aqueous trifluoroacetic acid and solvent B was 0.08% trifluoroacetic acid in acetronitrile. The different proteins were collected and digested with trypsin and analyzed by MALDI-TOF/MS.

### Patient selection and TMA

Breast cancer TMAs were purchased from Shanghai Hujing Biotech Co., Ltd, China and Cybrdi Inc., USA, which contained duplicates of 194 human breast cancer tissues and 15 normal breast tissues with a diameter of 1.0 mm. In addition, we collected 13 atypical hyperplasia of breast tissues and 18 normal breast tissues between 2007 and 2010 from the Cancer Institute (Hospital), Chinese Academy of Medical Sciences, Peking Union Medical College, Beijing, China. Ethical approval was provided by the Chinese Academy of Medical Sciences Cancer Hospital Ethics Committee. None of the patients received any neoadjuvant therapy prior to surgery. Prior patient consent and approval from the Institute Research Ethics Committee were obtained before we used these clinical materials for research purposes. Samples of normal tissues were taken from morphologically normal areas surrounding diseased tissue. Patients who received chemotherapy or radiotherapy before surgery were excluded. Samples with missing and invalid dots were also excluded. Therefore, in this study 168 female breast cancer patients (ranging in age from 31 to 82, with a mean of 58 years), 13 atypical hyperplasia and 33 normal breast tissues were analyzed. All surgically obtained tissues were fixed in 4% formalin and processed routinely. The tumors were classified according to the Pathological Tumor-Node-Metastasis (pTNM) system (sixth edition). The pathologic features examined included histological subtype, tumor size, primary tumor stage and regional lymph node involvement. The microscopic slides from all specimens were reviewed by at least two pathologists and one American Society for Clinical Pathology certified specialist in immunology. Table II summarizes the clinical pathological parameters analyzed in this study.

### IHC analysis

IHC was performed on 5 μm thick paraffin sections of tissues and breast cancer TMA. All slides were deparaffinized in xylene and rehydrated in graded alcohols. For antigen retrieval, slides were immersed in 0.01 M citrate buffer, pH 6.0, and boiled for 10 min in a microwave oven. Endogenous peroxidase was blocked in 0.3% H_2_O_2 _in PBS for 20 min. To reduce non-specific binding, sections were pre-incubated in normal goat serum. The anti-ATP synthase α-subunit mouse monoclonal antibody (mAb) (7H10-BD4, 1 μg/ml, Invitrogen, A21350) was incubated on the tissues overnight in a humidified chamber at 4°C. Subsequently, incubation of the samples with anti-mouse peroxidase-conjugated antibody was performed at room temperature for 30 min using a Power Vision Homo-Mouse IHC kit (ImmunoVision Technologies, Daly City, CA). Hematoxylin (Sigma) was used for counterstaining. Positive controls were included in each staining series. ATP synthase α-subunit immunopositivity was scored as follows: 0, no staining or sporadic staining in < 5% of tumor cells; 1, weak and sporadic staining in 5-25% of tumor cells; 2, weak staining in 26-50% of tumor cells; 3, strong diffuse cytoplasmic and membrane staining in 26-50% of tumor cells; and 4, strong, diffuse cytoplasmic staining and membrane staining in > 50% of tumor cells. For statistical analysis, negative (0), intermediate (1+ and 2+) and strongly (3+ and 4+) stained groups were created. Samples were evaluated under light microscopy by two independent pathologists and one American Society for Clinical Pathology certified specialist in immunology without prior knowledge of the patients' clinical data.

### Immunofluorescence analysis of breast cell lines

MDA-MB-231 and MCF-10F cells were grown on glass cover slips and stained with the anti-ATP synthase α-subunit antibody as described previously [24]. Briefly, MDA-MB-231 and MCF-10F cells were washed with PBS several times before fixation with 4% Para formaldehyde. A sample was permeated with 100% ethanol for 5 min at room temperature as a positive control. Cells were then incubated overnight with the anti-ATP synthase α-subunit mouse mAb (1 μg/ml) at 4°C and then washed twice in PBS. Subsequently, immunostaining was carried out for 1 h in the dark with a secondary antibody against mouse IgG2a conjugated to Alexa Fluor 488 (1:200, Molecular Probes) in staining buffer. Samples stained with the isotype mouse IgG2b antibody served as negative controls. The nuclei were labeled with PI (Propidium iodide). After final washings, the cells were mounted and analyzed using a Zeiss LSM-410 (Switzerland) confocal microscope at a magnification of 600×.

### Flow cytometry analysis of breast cell lines

MCF-7, MCF-7H, MDA-MB-453, MDA-MB-231 and MCF-10F cells were incubated at 20°C for 1 h in PBS, pH 7.4, containing 1% bovine serum albumin (BSA) plus primary mAbs in order to analyze cell-surface expression of ATP synthase α-subunit. The cells were then washed in PBS plus 1% BSA and incubated at 20°C for 30 min with a goat antibody against mouse IgG conjugated to Alexa 488 (1:200) and washed three times. A mouse IgG antibody served as negative controls. Propidium iodide was added to exclude the dead cells before analysis on a Coulter XL 4C flow cytometer (Beckman-Coulter).

### Cell proliferation assay

MDA-MB-231 were plated at a density of 5,000 cells/well in DMEM media depleted of FBS overnight to allow the cells to become quiescent, and MCF10F cells were likewise plated in Ham's F12 medium supplemented only with 20 μg/ml of epidermal growth factor [25]. Fresh media were added to the wells along with the anti-ATP synthase α-subunit antibody Mab9E10 (10 μg/ml, 50 μg/ml, 100 μg/ml). After every 24 h, 10 μl CCK-8 (Dojindo, Kumamoto, Japan) were added to the cells, which were then incubated for 2 h at 37°C. The absorbance, used to calculate the percent proliferation of the cells, was measured on a Thermo max plate reader at a wavelength of 450 nm according to the manufacturer's specifications. The MAb9E10 functional mAb generated against cell surface ATP synthase α-subunit was reported previously [26]. Briefly, hybridoma cells secreting mAb against ATPase were produced by polyethylene glycol-mediated fusions and screened by ELISA. Specificity of the mAb was demonstrated by immunofluorescence and confocal imaging, as well as flow cytometry analysis.

### Cell migration and invasion assays

Invasion assays were performed in trans well membrane filter inserts in 24-well tissue culture plates (BD Labware, Bedford, MA) as described previously [12]. Briefly, the trans well membrane filter inserts in a 24-well tissue culture plate contained 6.5 mm diameter, 8- μm pore size, 10-nm thick polycarbonate membranes. The lower surface of the porous membrane was coated with 10 μg/ml human plasma fibronectin at 4°C overnight and then blocked with 0.1% heat-inactivated BSA (Calbiochem, San Diego, CA) at 37°C for 45 min. MDA-MB-231 and MCF-10F cells were detached at 90% confluence with 2 mM EDTA/PBS, washed once in PBS and re-suspended in serum-free DMEM containing 0.1% BSA. A 300 μl cell suspension was added to the upper side of the inserts at a density of 3 × 10^4 ^cells/insert before anti-ATP synthase α-subunit primary mAb (100 μg/ml) and negative control mIgG (100 μg/ml) were incubated with the cells for 30 min. DMEM containing 1% FBS was added to the lower wells. Invasion was allowed to proceed at 37°C for 8 and 24 h. Cells that did not migrate through the filters were removed using cotton swabs, and cells that migrated through the inserts were fixed and stained with Trypan Blue. The number of migrated cells per insert was counted under a light microscope at magnification 20×. Experiments were carried out in triplicate and repeated at least three times. Data from several independent experiments were pooled and analyzed using a two-tailed, Student's t test.

### RT^2 ^Profiler PCR array

SABioscience Human Apoptosis PCR Array PAHS-3012 (Qiagen China, Shanghai, China) is an apoptosis pathway-focused gene expression profiling using real-time PCR. The system allows one to identify genes involved in apoptosis and programmed cell death. The array includes the TNF ligands and their receptors; members of the bcl-2 family, BIRC (baculoviral IAP repeat) domain proteins, CARD domain (caspase recruitment domain) proteins, death domain proteins, TRAF (TNF receptor-associated factor) domain proteins and caspases. RNA was isolated using RNA easy Mini kit (Qiagen). The single strand cDNA from 1 mg total RNA was synthesized using RT^2 ^First Strand Kit (SABioscience). Real-time PCR was performed according to the user manual of RT^2 ^Profiler PCR array system (SABioscience) using SYBR Green PCR Master Mix in a Light Cycler 480 system (Roche Diagnostics). Data were analyzed using Excel-based PCR Array Data Analysis Templates (SABioscience). Three samples were analyzed: Control group, MDA-MB-231 cells treated with IgG for 24 hours; Test group 1, MDA-MB-231 cells treated with MAb9E10 100 mg/ml for 24 hours; Test group 2, MDA-MB-231 cells treated with MAb9E10 100 mg/ml for 48 hours.

### Statistical analysis

The breast tissue array data were transferred to a PC and statistically analyzed using SPSS Version 10 for Windows (SPSS Inc., Chicago, IL). Correlation of the ATP synthase expression between the several different clinic pathological factors was calculated with the non-parametric Spearman correlation coefficient. Results with a *P *< 0.05 were deemed statistically significant.

## Results

### ATP synthase α-subunit is over-expressed in the highly invasive MCF-7-H subline

**Figure 1:**
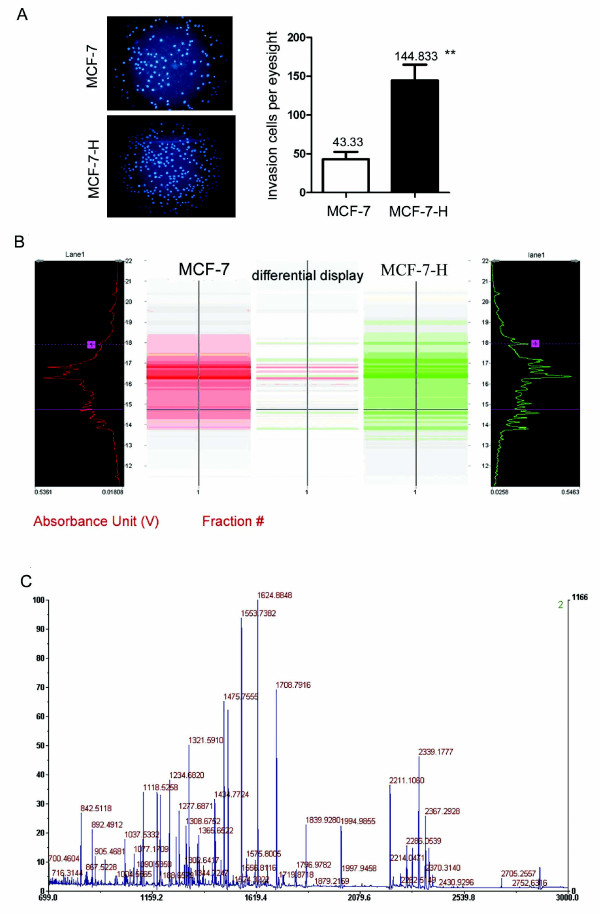
**Identification of ATP synthase α-subunit as a differentially expressed protein between MCF-7-H and MCF-7 cells by PF 2D and MALDI-TOF-MS**. ***A***, The sub-line MCF-7-H was selected from MCF-7 cells for high invasive ability using the trans well invasion assay, cells were stained with DAPI and the asterisks ** indicate P < 0.01. ***B***, The display map of differentially expressed proteins between the MCF-7-H and MCF-7 cell lines. The image was analyzed by ProteoVue and DeltaVue software. ProteoVue allows comparison of multiple or all second dimension runs for one sample in a 2-D map using either gray scale or a color-coded format, where color hue or its intensity is proportional to the relative quantitative UV intensity of each peak. The DeltaVue software quantitatively displays one protein map in shades of red and the other map in shades of green. The difference between the two maps is obtained by point-by-point subtraction or by area difference and displayed as a third map in the middle. The color (red or green) at a particular location in the difference map indicates which protein is more abundant, and the color brightness indicates the quantitative difference. ***C***, MALDI-TOF mass spectrum of ATP synthase α-subunit after trypsin digestion. MS-Digest search using the peptide mass fingerprint data indicated that 10 peptides were matched with peptides from ATP synthase α-subunit, giving sequence coverage of 28% (126/450 aa) of the protein.

To establish an breast carcinoma invasion model, MCF-7 cells were seeded onto Matrigel-coated trans wells. The cells were allowed to invade into the lower chamber of the trans well, collected, expanded and then re-seeded onto another Matrigel-coated trans well. Cells were harvested after four rounds of selection, resulting in the establishment of a relatively stable, highly invasive subline, MCF-7-H. The invasive ability of this line was about 3.3-fold greater than that of the MCF-7 parent cell line (Figure 1A) in Matrigel invasion tests, but equal to that of the third selection round line, suggesting that the metastatic potential of the subline reached a plateau by the fourth round of selection. Thus, by Matrigel invasive subpopulation selection, we were able to establish the highly invasive and metastatic breast cancer cell line MCF-7-H.

### 2-D liquid chromatography and MALDI-TOF MS analysis

Image analysis of the 2-D map revealed 72 protein peaks with significantly differential expression between MCF-7-H and MCF-7 cells, and 9 of these protein peaks were further identified by tryptic digestion, peptide mass fingerprinting and MS. Five proteins were down-regulated and four proteins up-regulated in MCF-7-H cells, including ATP synthase α-subunit. The display map of differentially expressed proteins between the MCF-7-H and MCF-7 cell lines and the MALDI-TOF/MS tryptic peptide mass map of ATP synthase α-subunit are shown in Figure 1B and 1C, respectively. MS-Digest search using the peptide mass fingerprint data indicated that 10 peptides were matched with peptides from ATP synthase α-subunit, giving sequence coverage of 28% (126/450 aa) of the protein. The amino acid sequence of this protein can be accessed in the National Center for Biotechnology Information (NCBI) protein database under NCBI Accession # 4757810.

### IHC and TMA analysis of ATP synthase α-subunit

**Figure 2:**
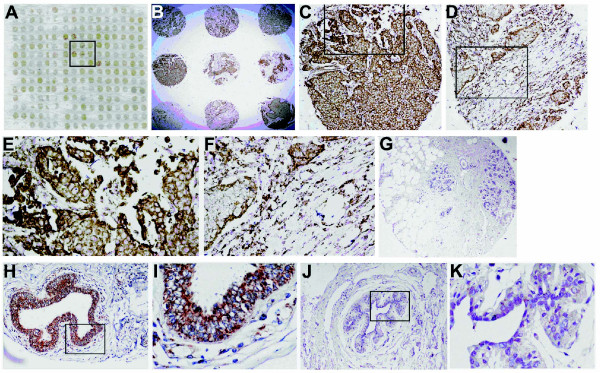
**IHC analysis of ATP synthase α-subunit expression in breast cancer tissue, atypical breast hyperplasia and normal breast tissue**. The breast cancer tissue microarrays were probed with the anti-ATP synthase α-subunit mAb (*brown*), and hematoxylin was used for counterstaining (*blue*). ***A***, A representative arrangement of "spots" on the breast cancer tissue microarray slide. ***B***, Low-power magnification shows strong and moderate immunoreactivity samples on the microarray slide (40×). ***C ***and ***D***, Examples of a strong and a moderate immunostaining, respectively, of ATP synthase α-subunit in epithelial cells of breast infiltrating duct carcinoma samples at increased magnification (200×). ***E ***and ***F***, A high-power view suggests immunoreactivity of the ATP synthase α-subunit antibody in the cellular membrane and granular cytoplasmic distribution (400×). ***G***, Absent staining in normal breast tissue. Original magnification (200×). ***H, I, J ***and ***K***, Atypical breast hyperplasia exhibiting a less intense (H and I) or absent immunostaining (J and K) at low-power (H and J, 200×) and high-power (I and K, 400×) magnifications.

IHC staining was performed on 214 cases in total. In all 33 normal breast epithelia, a weak or missing ATP synthase α-subunit signal was detected at the cell membrane and in the cytoplasm. The overall percentage of atypical hyperplasia breast tissues staining positive for ATP synthase α-subunit was 23.1% (3/13), while that in breast cancer tissues was 94.6% (159/168) (Table 1). Detection of the ATP synthase α-subunit was strong in 98 breast cancer patients (58.3%), moderate in 61 patients (36.3%) and negative in 9 patients (5.3%) (Table 2). The staining pattern was predominantly at the cellular membrane and cytoplasm (Figure 2E and 2F). The cytoplasmic immunoreactivity was granular and appeared in epithelial cells. Figure 2 shows an example of the tissue array analysis for ATP synthase α-subunit in breast cancer samples, atypical breast hyperplasia and normal breast tissue. Expression of the ATP synthase α-subunit in breast cancer (Figure 2C and 2D) was significantly higher than in breast atypical hyperplasia and normal breast tissues (Figure 2G, H, I, J and 2K). The analysis showed that the higher ATP synthase α-subunit expression was significantly associated with tumor size, histological grade and stage (*P *< 0.05). No statistically significant differences were found among cases with different ages or lymph node invasion (*P *> 0.05) (Table 2). Analysis of gene expression data sets from a cancer gene microarray meta-analysis public database [27]http://www.oncomine.org revealed that ATP synthase α-subunit mRNA expression is markedly higher in carcinoma cell lines than in normal tissues and cell lines (Additional file 1).

**Table 1:** ATP synthase expression in normal, atypical breast hyperplasia and breast cancer tissues

	N	ATP synthase expression		*P *value
		Negative	Positive	
Normal	33	26	7 (21.2%)	< 0.001
Hyperplasia	13	10	3 (23.1%)	
breast cancer	168	9	159 (94.6%)	

*P *value of chi-square analysis.

**Table 2:** Clinic pathological data and ATP synthase α-subunit expression in 168 breast cancers

	α-ATP synthase expression			Total	*P *value
			
	Negative	Moderate	Strong		
**Age**					

< 50	5 (7.0%)	30 (42.3%)	36 (50.7%)	71(100%)	Not significant

≥ 50	4 (4.1%)	31 (32.0%)	62 (63.9%)	97 (100%)	

**Size**					

< 2 cm	2 (13.3%)	10 (66.7%)	3 (20.0%)	15 (100%)	*P *< 0.05

≥ 2 cm	2 (3.6%)	24 (42.8%)	30 (53.6%)	56 (100%)	

**Histological grade**					

Well differentiated	3 (12.5%)	11 (45.8%)	10 (41.7%)	24 (100%)	*P *< 0.05

Moderately differentiated	6 (5.3%)	43 (37.7%)	65 (57.0%)	114 (100%)	

Poorly differentiated	0 (0.0%)	3 (15.0%)	17 (85.0%)	20 (100%)	

**TNM Stage**					

I	2 (22.2%)	4 (44.5%)	3 (33.3%)	9 (100%)	*P *< 0.05

II	2 (4.6%)	24 (54.5%)	18 (40.9%)	44 (100%)	

III	0 (0.0%)	3 (23.1%)	10 (76.9%)	13 (100%)	

**Lymph node invasion**					

Positive	3 (5.9%)	18 (35.3%)	30 (58.8%)	51 (100%)	Not significant

Negative	2 (4.8%)	11 (26.2%)	29 (69.0%)	42 (100%)	

*P *value of chi-square analysis.

### Analysis of ATP synthase α-subunit surface expression on breast cancer cells by immunofluorescence microscopy and flow cytometry

**Figure 3:**
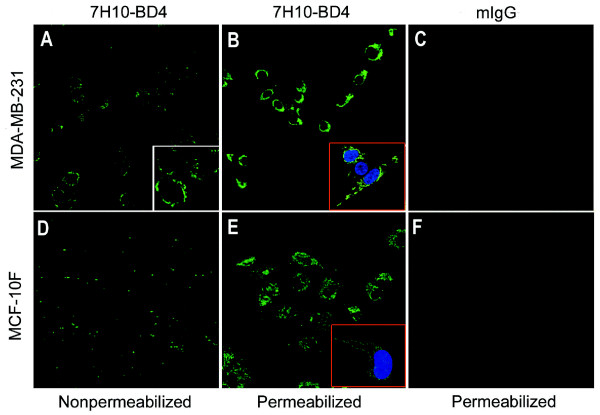
**Immunofluorescence localization of the ATP synthase α-subunit on the surface of breast cancer cells by confocal microscopy**. Cells were stained with an α-ATP synthase antibody, followed by secondary antibody, as detailed in Materials and Methods. ***A ***and ***D***, Non-permeabilized MDA-MB-231 and MCF-10F immunostained with a murine mAb specific for the ATP synthase α-subunit(600×). ***B ***and ***E***, Image obtained from MDA-MB-231 and MCF-10F cells permeabilized with ethanol (100%). ***C ***and ***F***, Control experiments for antibody specificity using isotypic purified mouse IgG in cells(600×).

**Figure 4:**
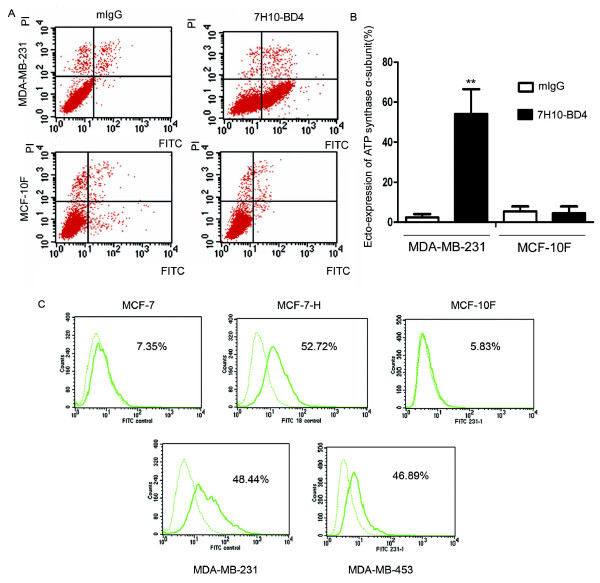
**Detection of ATP synthase α-subunit on the surface of breast cancer cells by flow cytometry**. MCF-7, MCF-7H, MDA-MB-453, MDA-MB-231 and MCF-10F cells were analyzed by flow cytometry as described in Materials and Methods. Histogram plots are shown of cells, determined to be intact by propidium iodide exclusion, which were labeled with 7H10-BD4 mAb for the α-subunit of ATP synthase or control mouse IgG. Significantly higher ecto-expression of ATP synthase α-subunit was found in MCF-7H, MDA-MB-453, MDA-MB-231 cells compared with control IgG staining. No significant ecto-expression of ATP synthase α-subunit in MCF-7 and MCF-10F cells was observed compared with the control.

Immunofluorescence microscopy analysis using a specific antibody confirmed the surface localization of the ATP synthase α-subunit on non-permeabilized MDA-MB-231 cells (Figure 3A). Control experiments were performed with isotype mouse IgG (Figure 3C, F) and permeabilized cells in the presence of the anti-α-subunit ATP synthase antibody (Figure 3B, E). While the permeabilized normal breast cell line MCF-10F (Figure 3E) also reacted with the anti-ATP synthase α-subunit antibody, no specific reactivity was observed with non-permeabilized MCF-10F cells (Figure 3D). Surface expression of ATP synthase on MDA-MB-231 and MCF-10F cells were further assessed by flow cytometry. The results showed a significantly higher proportion of MDA-MB-231 cells (54.1 ± 12.4%) with ecto-expression of the ATP synthase α-subunit compared with the IgG isotype control group (2.4 ± 1.7%) (*P *< 0.01, Figure 4A, B). No ecto-expression of the ATP synthase α-subunit was detected in MCF-10F cells (4.5 ± 3.4%) when compared with its control group (5.4 ± 2.5%) (*P *> 0.05, Figure 4A, B). We also detected the ecto-expression of ATP synthase α-subunit in other three breast cell lines, and the ecto-expression is from 5.83% to 52.72% in these cell lines (Figure 4C)

### Anti-proliferative effect of anti-ATP synthase α-subunit antibody on breast cancer cells

**Figure 5:**
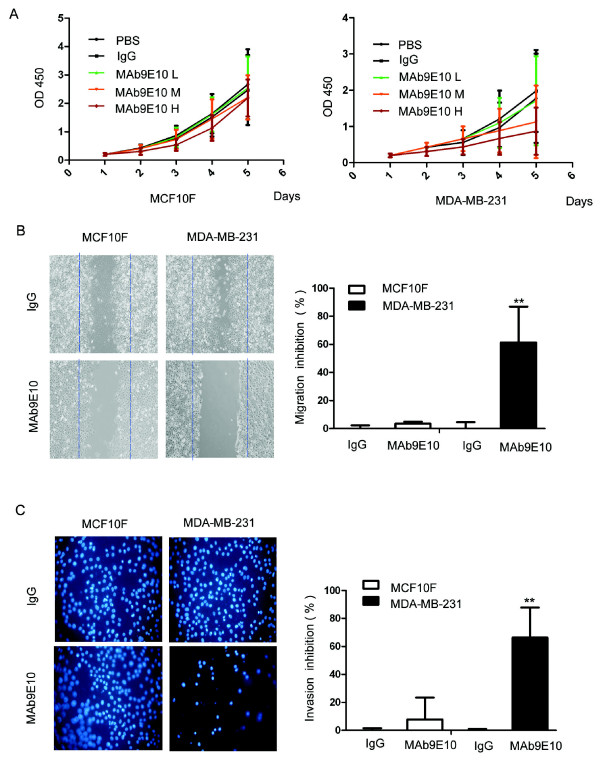
**Inhibition of breast cancer cell proliferation, migration and invasion by a functional anti-ATP synthase α- subunit antibody**. ***A ***, MDA-MB-231 and MCF-10F were plated at a density of 5,000 cells/well in serum-free media for overnight fasting. The α-subunit of ATP synthase was added at concentrations of 10 μg/ml (low dose), 50 μg/ml (medium dose) or 100 μg/ml (high dose). CCK-8 solution was added after 24 h, and the absorbance used to calculate percent proliferation was measured on a Thermo max plate reader at a wavelength of 490 nm. Growth of MDA-MB-231 cells treated with high dose MAb9E10 was significantly suppressed compared with IgG control treatment. MCF-10F cell proliferation was not inhibited by MAb9E10 treatment. ***B***, Migration of MDA-MB-231 and MCF-10F cells were measured using wound-healing assays. MDA-MB-231 cells treated with MAb9E10 100 μg/ml showed lower migration ability comparing with mIgG control; ** *P *< 0.01. No significant inhibition of migration was observed when MCF-10F cells were treated with MAb9E10. ***C***, Invasion of MDA-MB-231 cells was measured using transwell inserts coated with fibronectin (10 μg/ml). After treatment with anti-ATP synthase α-subunit antibody or control mIgG, cells that migrated through the filters onto the lower surface were fixed, stained and photographed (200×). In each individual experiment, the invaded cells were counted from at least three randomly selected fields. Results were averaged from at least three individual experiments. MDA-MB-231 cells treated with MAb9E10 100 μg/ml also showed lower invasion ability comparing with mIgG control (Figure 5C). Almost no inhibition of invasion when MCF-10F cells treated with MAb9E10. The bar graph displays means ± S.D; ** *P *< 0.01.

To gain insight into the function of ATP synthase in breast cancer and test whether ATP synthase represents a potential therapeutic target, we used MAb9E10, an anti-ATP synthase α-subunit mAb produced as previously described [26]. After blockade of surface ATPase with this mAb on human breast adenocarcinoma MDA-MB-231 cells, an ATP determination kit and CellTiter96 AQueous Assay (MTS) assay were used to detect the effect of the antibody on extracellular ATP modification and cell proliferation. Results showed that MAb9E10 inhibited the MDA-MB-231 cell proliferation in a concentration-dependent manner. The growth of MDA-MB-231 cells treated with MAb9E10 was significantly suppressed compared with that of cells treated with IgG control. The proliferation of cells (OD450 = 0.87 ± 0.65) treated with MAb9E10 in the high dose group (100 μg/ml) was significantly lower than that of the mIgG control group (OD450 = 1.98 ± 1.13) (Figure 5A). There was no inhibition of proliferation when MCF-10F cells were treated with high dose (100 μg/ml) MAb9E10 (OD450 = 2.19 ± 0.65), which was nearly equal to that of the control group (OD450 = 2.47 ± 1.23) (Figure 5A).

### Inhibition of MDA-MB-231 migration and invasion by anti-ATP synthase α-subunit antibody

To investigate the effect of ATP synthase expression on cell motility and whether ATP synthase is involved in the progression and metastasis of breast cancer, the MDA-MB-231 and MCF-10F metastatic breast cancer cell lines were then tested for their directional motility on fibronectin and for their invasion ablity in a chemohaptotactic invasion assay. MDA-MB-231 cells treated with MAb9E10 (100 μg/ml) showed lower migration ability (66.2% migration inhibition) compared with IgG control treatment (*P *< 0.01; Figure 5B). There was no significant inhibition of migration (7.8%) when MCF-10F cells were treated with MAb9E10 at the same dose (*P *> 0.05; Figure 5B). In trans well assays, MDA-MB-231 cells treated with MAb9E10 (100 μg/ml) also showed a lower invasion ability (61.3% invasion inhibition) compared with IgG control treatment (*P *< 0.01; Figure 5C). There was also no significant inhibition of invasiveness of MCF-10F cells treated with MAb9E10 at the same dose (3.4%) vs. the mIgG control group (*P *> 0.05; Figure 5C).

### Anti-ATP synthase α-subunit antibody regulated apoptosis pathway of MDA-MB-231 cells

**Figure 6:**
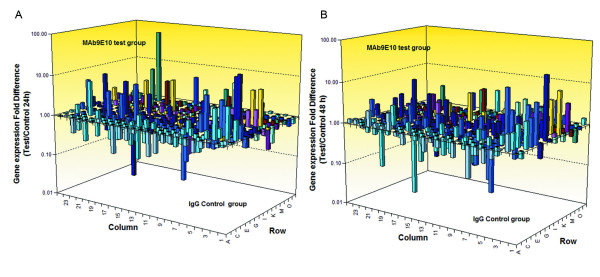
**Anti-ATP synthase α-subunit antibody regulated apoptosis pathway of MDA-MB-231 cells**. Gene's expression of MDA-MB-231cells was measured using SABioscience Human Apoptosis PCR Array PAHS-3012. Three samples were analyzed: Control group, MDA-MB-231 cells treated with IgG for 24 hours; Test group 1, MDA-MB-231 cells treated with MAb9E10 100 mg/ml for 24 hours; Test group 2, MDA-MB-231 cells treated with MAb9E10 100 mg/ml for 48 hours. Calculate the ^Δ^Ct for each pathway-focused gene in each treatment group. ^Δ^Ct (group) = average Ct - average of HK genes' Ct for group array. Calculate the ^ΔΔ^Ct for each gene across two PCR Arrays (or groups). ^ΔΔ^Ct = ^Δ^Ct (Test group) - ^Δ^Ct (Control group). Calculate the fold-change for each gene from Test group to control group as 2^-ΔΔCt^. A, Gene expression fold difference between Test group 1 and Control group. B, Gene expression fold difference between Test group 2 and Control group.

In order to identify apoptosis and/or programmed cell death molecules implicated into the treatment with anti-ATP synthase α-subunit antibody, we used the SABioscience Human Apoptosis PCR Array PAHS-3012. We analyzed the expression of 370 key genes involved in apoptosis, or programmed cell death with this PCR Array (Figure 6A, B). This array includes the TNF ligands and their receptors; members of the bcl-2 family, BIRC (baculoviral IAP repeat) domain proteins, CARD domain (caspase recruitment domain) proteins, death domain proteins, TRAF (TNF receptor-associated factor) domain proteins and caspases. Using this real-time PCR, we can easily and reliably analyze the expression of a focused panel of genes related to apoptosis with this array. Comparison of PCR results between Test group and control group showed that 23 genes were significantly up-regulated and 18 genes were significantly down-regulated both in test group1 and 2 (fold changes Table 3 and Table 4). The raw data of PCR array was attached in additional file 2. Results showed that treated with Anti-ATP synthase α-subunit antibody, a lot of apoptosis induced genes were up-regulated: FOXO1 (30.64), CASP9 (17.37) and CASP7 (4.43). A lot of anti-apoptotic genes were down-regulated at the same time: CARD9 (-34.63), CCL2 (-16.47) and BIRC3 (-9.66). These data could partially explain the function of Anti-ATP synthase α-subunit antibody.

**Table 3:** Up regulated genes of MDA-MB-231 cells treated with MAb9E10 antibody 24 and 48 hours (Fold change)

	Symbol	UniGene	Description	24 h group	48 h group
1	FOXO1	Hs.370666	Forkhead box O1	17.95	30.64
2	CASP9	Hs.329502	Caspase 9, apoptosis-related cysteine peptidase	2.67	17.37
3	NDUFS3	Hs.502528	NADH dehydrogenase (ubiquinone) Fe-S protein 3, 30 kDa (NADH-coenzyme Q reductase)	7.48	10.73
4	HSPA1B	Hs.274402	Heat shock 70 kDa protein 1B	7.68	9.62
5	COL4A3	Hs.570065	Collagen, type IV, alpha 3 (Goodpasture antigen)	2.23	8.86
6	NFKB1	Hs.654408	Nuclear factor of kappa light polypeptide gene enhancer in B-cells 1	6.71	6.3
7	F2R	Hs.482562	Coagulation factor II (thrombin) receptor	6.59	5.38
8	CRYAB	Hs.408767	Crystallin, alpha B	6.62	4.97
9	SOCS2	Hs.485572	Suppressor of cytokine signaling 2	4.09	4.89
10	NOD2	Hs.592072	Nucleotide-binding oligomerization domain containing 2	6.46	4.88
11	PIK3R2	Hs.371344	Phosphoinositide-3-kinase, regulatory subunit 2 (beta)	1.72	4.75
12	PRKCE	Hs.580351	Protein kinase C, epsilon	2.83	4.66
13	CASP7	Hs.9216	Caspase 7, apoptosis-related cysteine peptidase	4.85	4.43
14	TBX3	Hs.714737	T-box 3	3.6	4.24
15	BIRC8	Hs.348263	Baculoviral IAP repeat containing 8	2.88	4.08
16	PIM2	Hs.496096	Pim-2 oncogene	4.23	3.79
17	HSPB1	Hs.520973	Heat shock 27 kDa protein 1	1.75	3.76
18	IL31RA	Hs.55378	Interleukin 31 receptor A	3.46	3.46
19	MADD	Hs.82548	MAP-kinase activating death domain	2.06	3.43
20	PEA15	Hs.517216	Phosphoprotein enriched in astrocytes 15	7.8	3.38
21	BNIP1	Hs.145726	BCL2/adenovirus E1B 19 kDa interacting protein 1	5.48	3.28
22	CASP14	Hs.466057	Caspase 14, apoptosis-related cysteine peptidase	7.09	3.09
23	MAL	Hs.80395	Mal, T-cell differentiation protein	2.42	3.04

**Table 4:** Down regulated genes of MDA-MB-231 cells treated with MAb9E10 antibody 24 and 48 hours (Fold change)

	Symbol	UniGene	Description	24 h group	48 h group
1	CARD9	Hs.694071	Caspase recruitment domain family, member 9	-3.18	-34.63
2	CCL2	Hs.303649	Chemokine (C-C motif) ligand 2	-5.91	-16.47
3	CUL3	Hs.372286	Cullin 3	-20.23	-10.06
4	BIRC3	Hs.127799	Baculoviral IAP repeat containing 3	-3.96	-9.66
5	IL18	Hs.83077	Interleukin 18 (interferon-gamma-inducing factor)	-2.05	-8.89
6	TP63	Hs.137569	Tumor protein p63	-3.43	-8.6
7	SPP1	Hs.313	Secreted phosphoprotein 1	-2.88	-7.61
8	TNFRSF21	Hs.443577	Tumor necrosis factor receptor superfamily, member 21	-3	-6.72
9	F2	Hs.655207	Coagulation factor II (thrombin)	-35.19	-5.9
10	AIFM3	Hs.163543	Apoptosis-inducing factor, mitochondrion-associated, 3	-1.56	-5.79
11	CARD8	Hs.446146	Caspase recruitment domain family, member 8	-3.07	-5.52
12	PMAIP1	Hs.96	Phorbol-12-myristate-13-acetate-induced protein 1	-4.52	-4.7
13	CD27	Hs.355307	CD27 molecule	-2.63	-4.34
14	HMGB1	Hs.593339	High mobility group box 1	-1.74	-4
15	INHA	Hs.407506	Inhibin, alpha	-5.02	-3.75
16	TP53I3	Hs.50649	Tumor protein p53 inducible protein 3	-1.89	-3.74
17	BRCA1	Hs.194143	Breast cancer 1, early onset	-1.99	-3.49
18	MYBL2	Hs.179718	V-myb myeloblastosis viral oncogene homolog (avian)-like 2	-2.42	-3.32

## Discussion

In this study we combined several advanced techniques, such as proteomic analysis using the Proteome Lab PF 2D fractionation system, MALDI-TOF/MS and tissue microarray, with functional analysis to identify novel biomarkers for improved prediction of progression, metastasis and response to therapy for breast cancer. The work was also performed in an attempt to better understand molecular mechanisms involved in breast cancer carcinogenesis and metastasis. Our studies revealed one highly over-expressed protein, the α-subunit of ATP synthase, in breast cancer. This observation was validated, refined and extended through a series of IHC on tissue microarray, immunofluorescence and functional analyses. The over-expression of ATP synthase α-subunit was detected by IHC in several different human tumor samples, including breast cancer, hepatocellular carcinoma, colon cancer and prostate cancer (data not shown). Since ATP synthase α-subunit was highly over-expressed in 94.6% of breast cancer samples tested while being undetectable in normal breast tissues, this study was focused on the expression, functional implication and potential involvement of ATP synthase in the progression and metastasis of breast cancer. Levels of ATP synthase α-subunit expression were strongly correlated with large tumor size, poor tumor differentiation and advanced stages of tumor. High expression of ATP synthase in breast cancer compared to normal breast tissue and hyperplasia indicates that it can serve as a biomarker in screening, diagnosis and prediction of breast cancer progression. ATP synthase expression may also be useful when used in combination with more traditional pathologic indicators such as tumor size, TNM stage and tumor grade to ascertain the prognosis of breast cancer patients.

Prior studies using DNA arrays relied on the expression of gene transcript in cancers, with little focus on the detection of the protein [28]. With the recent development of similar technologies at the DNA and protein levels and several studies demonstrating the clinical potential of the proteomic approach, there is no reason to limit profiling to RNA. Proteomic analysis has the potential to complement and further enlarge the wealth of information generated by genomics for several reasons. Levels of mRNA levels do not necessarily correlate with corresponding protein abundance, as additional complexity is conferred by protein post-translational modifications, including phosphorylation, acetylation and glycosylation, or protein cleavage. These modifications are not detectable at the mRNA level but play significant roles in protein stability, localization, interactions and functions. Finally, proteins represent more accessible and relevant therapeutic targets than nucleic acids. Thus, we surveyed primary breast cancers for expression of ATP synthase by immunostaining with the mouse anti-α-subunit ATP synthase antibody and found a high frequency of immunoreactivity.

During energy generation in the mitochondria, the ATP synthase is driven by a gradient of protons across a membrane, produced by the organelle's oxygen-burning metabolism [13]. However, in a low oxygen or acidosis microenvironment, where mitochondria ATP synthase levels are very low, the cell surface ATP synthase of tumor cells may manufacture the ATP using the gradient from the inside of cells compared to the outside, which could result from the lack of oxygen [29-31]. When mitochondria are in low oxygen conditions or matrix pH decreases, the proton gradient favorable for ATP synthesis declines [32]. Therefore, it may be more feasible for the cell in such an environment to produce ATP on its surface. For example, ATP synthase shows greater activity under conditions of acidosis, a hallmark of the tumor microenvironment [33]. Cell-surface-generated ATP may be transported into the cell to provide a source of energy in the tumor microenvironment where intracellular levels are very low. It is hypothesized that certain lethal tumor phenotypes, such as invasion and metastasis, arise not only from genetic alterations within the tumor but from tumor microenvironmental stimuli, such as acidosis. Endothelial and tumor cells could also have a plentiful supply of ADP for conversion to ATP to provide cancer cells with an extra energy source for survival since red blood cells release high levels of ADP in low-oxygen conditions.

Cellular invasiveness is an enhanced or aberrant cell movement and a major characteristic of poorly differentiated cancers [34]. In metastatic cancers, cells usually acquire the ability to invade surrounding tissues and then metastasize to secondary lesions. Therefore, the aberrant motile feature of cancer cells is responsible for the initial phase of cancer metastasis, and cell motility or invasiveness is one of the multiple steps that can be inhibited by a cancer metastasis suppressor. ATP synthase may also play critical roles in tumor cell metastases. It was previously identified as the binding target of angiostatin, a potent antagonist of angiogenesis and the growth of tumor cell metastases [16,35,36]. F1-F0-ATP synthase constitutes the major (endothelial cells) EC-binding site for angiostatin, which is active in ATP synthesis and inhibits the endothelial cells proliferation by blocking conformational changes of the enzyme complex required for ATP synthesis or hydrolysis. ATP synthase activities of the enzyme are inhibited by angiostatin as well as by antibodies directed against the α- and β-subunits of ATP synthase in cell-based and biochemical assays. These experimental results suggest that the inhibitors of ATP synthase activity, such as antibodies against ATP synthase, function as antagonists of angiogenesis. Recent studies also suggested that the polyclonal or monoclonal antibodies against the β-catalytic subunit of F1F0 ATPase could efficiently inhibit the activities of ecto-F1F0 ATPase. Wang et al. [37] demonstrated inhibition of tumor growth in a hepatoma xenograft mouse model using a monoclonal antibody (mAb6F2C4) against the catalytic β subunit of ATP synthase. In accordance with findings of Chi et al [38]. and Zhang et al [26] treating MDA-MB-231 cells and HUVECs with other monoclonal antibodies against the β subunit of ATP synthase the authors show that mAb6F2C4 inhibits extracellular ATP synthesis to a greater degree at low pH (6.7) than at normal pH (7.4).

In order to identify apoptosis and/or programmed cell death molecules implicated into the treatment with anti-ATP synthase α-subunit antibody, we analyzed the expression of 370 key genes involved in apoptosis, or programmed cell death with this PCR Array. PCR Arrays are the most reliable tools for analyzing the expression of a focused panel of genes. High-quality primer design and master mix formulation enable the PCR Array to amplify 384 different gene-specific products simultaneously under uniform cycling conditions. A lot of new genes were identified and these genes may relate with the function of Anti-ATP synthase α-subunit antibody.

In the present study, two breast tissue cell lines, MDA-MB-231, the highly invasive breast cancer cell line with a relatively higher level of cell surface ATP synthase α-subunit, and MCF10F, an immortalized (non-tumor derived) human breast epithelial cell line with no detectable level of cell surface ATP synthase, were selected to study the role of ATP synthase in cancer cell proliferation and migration. The anti-ATP synthase α-subunit antibody exerted a significant inhibitory effect on the proliferation and migration of the breast cancer cells. To our knowledge, our study showed for the first time that antibody mediated blockade of the ATP synthase α-subunit antibody can inhibit breast cancer cells and attenuate the directional cell migration of cancer cells *in vitro*. Although ATP synthase may also play critical roles in tumor cell metastases as the binding target of angiostatin in endothelial cells, a potent antagonist of angiogenesis and the growth of tumor cell metastases, our results showed that an anti-ATP synthase α-subunit antibody directly inhibited the tumor cell migration. Thus, decreased cell motility and migration likely play a major role in ATP synthase mediated cancer metastasis. The suppression of cell migration by the anti-ATP synthase α-subunit antibody was not limited to a specific ECM interaction.

## Conclusions

In summary, our results demonstrated that over-expression of ATP synthase α-subunit may be involved in the progression and metastasis of breast cancer, and it may represent a potential biomarker for diagnosis and a promising therapeutic target for anti-tumor and anti-metastasis therapy. The development of antibodies to ATP synthase in place of angiostatin mimetics may be advantageous due to increased specificity and affinity. An antibody against ATP synthase may ultimately prove useful, either alone or in combination with other treatments, to improve the effectiveness of breast cancer therapy.

## Abbreviations used

2-D: two-dimensional; MALDI-TOF/MS: matrix-assisted laser desorption/time-of-flight mass spectrometry; IHC: immunohistochemistry; ECM: extracellular matrix; FN: fibronectin; LN: laminin; pI: isoelectric point; pTNM: Pathological Tumor-Node-Metastasis; TMA: tissue microarray.

## Competing interests

The authors declare that they have no competing interests.

## Authors' contributions

PJ, SLC, TYF and ZZ performed the most of the experiments. LYP, LL, WJ, ZXM, WSY, ZYL, HSY, ZWL and FX coordinated data collection and quality control, and assisted in the interpretation of results. DXL participated in acquiring laboratory data analysis. NJ and LGL participated in study design and coordination, data analysis and interpretation and drafted the manuscript. All authors read and approved the final manuscript.
